# Comparison of exhaustion symptoms in patients with stress-related and other psychiatric and somatic diagnoses

**DOI:** 10.1186/s12888-019-2066-y

**Published:** 2019-03-04

**Authors:** Jesper Kristiansen, Maria Kristine Friborg, Nanna Eller, Lars Peter Andreas Brandt, David John Glasscock, Jesper Pihl-Thingvad, Roger Persson, Aniella Besèr, Marie Åsberg, Sannie Vester Thorsen

**Affiliations:** 1 National Research Centre for the Working Environment, Copenhagen, Denmark; 2 Department of Occupational and Environmental Medicine, Bispebjerg Hospital, Copenhagen, Denmark; 3 Department of Occupational and Environmental Medicine, Odense University Hospital, Odense, Denmark; 4 Occupational and Environmental Medicine, Department of Clinical Research, University of Southern Denmark, Odense, Denmark; 5 Danish Ramazzini Centre, Department of Occupational Medicine, The Regional Hospital West Jutland – University Research Clinic, Herning, Denmark; 6 Department of Psychology, Lund University, Lund, Sweden; 7 Department of Laboratory Medicine, Division of Occupational and Environmental Medicine, Lund University, Lund, Sweden; 8 Department of Clinical Sciences, Karolinska institutet at Danderyd Hospital, Stockholm, Sweden

**Keywords:** Stress, Exhaustion, Occupational health, Disorder

## Abstract

**Abstract:**

**Background:**

Several rating scales assessing stress-related symptoms of exhaustion have emerged in recent years. However, more knowledge is needed about the performance of these rating scales in patients with stress-related disorders as well as in other patient groups. With the recently developed Karolinska Exhaustion Disorder Scale (KEDS), we compared symptoms of exhaustion in different patient groups that were sorted according to diagnosis.

**Methods:**

Patients were sampled consecutively from departments of occupational medicine (DOM) at three Danish hospitals. The total study group comprised 698 care-seeking patients (487 women). Patients with stress-related diagnoses (*n* = 217; the International Classification of Diseases [ICD]-10 code F43: reaction to severe stress and adjustment disorder) were compared to a diverse group of patients with a range of somatic diagnoses (*n* = 338) and to patients with other psychiatric diagnoses (*n* = 143), including subgroups with major depression disorder (*n* = 34; F32 and F33) and problems related to employment and unemployment (*n* = 99; Z56). The data were analysed using linear mixed models with the SPSS statistical program.

**Results:**

The mean KEDS sum score in patients with stress-related diagnoses (29.3; SD = 8.0) was significantly higher than in patients with other psychiatric diagnoses (25.9; SD = 9.5) and in patients with somatic diagnoses (17.6; SD = 10.8). The subgroup with a major depression disorder had high mean KEDS sum scores (31.4, SD = 8.1), similar to patients with stress-related diagnoses, while the mean KEDS sum score in patients with problems related to employment and unemployment (Z56) was 23.5 (SD = 9.0). Young and old patients scored similarly on KEDS, but in patients with somatic diagnoses, female patients scored significantly higher than male patients.

**Conclusion:**

The symptoms of exhaustion measured with KEDS were higher in patients with stress-related diagnoses and major depression disorder than in patients with somatic diagnoses. The intermediate level of the symptoms of exhaustion that were associated with problems related to employment and unemployment, (Z56) compared to the lower level of the symptoms with somatic diagnoses, suggests that KEDS might be useful in detecting mild, prodromal states of exhaustion. This needs further investigation.

## Background

Stress-related health problems seem to be increasing in several counties [1–3]. Data from various sources indicate that stress-related health problems also constitute a significant problem in Denmark. For example, the number of mental disorders reported to the Danish Working Environment Authority almost doubled from 2011 to 2014, making mental disorders the second most frequently reported occupational disorder after musculoskeletal diseases [4]. These data, as well as other data [3, 5, 6], suggest that stress-related health problems are prevalent and have increased in recent years in Denmark. Successful prevention and treatment of stress-related health problems are urgently needed as well as instruments for assessment and monitoring of symptoms. To serve this purpose, a rating scale should be based on the core symptoms of stress-related disorders. However, the mental health problems that result from chronic stress are not recognized as a unitary psychiatric disease in Denmark, and therefore no common diagnostic criteria for stress-related disorders exist. This means that there is no agreement on the core symptoms that such an instrument should address. In contrast to this situation in Denmark, the National Board of Health and Welfare in Sweden has provided tentative diagnostic criteria for stress-related mental health problems and has suggested that the term *exhaustion disorder* and the ICD-10 diagnostic code F43.8A be used [7]. The core symptom of exhaustion disorder is physical and mental exhaustion, which manifests itself as fatigue, an increased need for recovery after mental exertion, and reduced mental and physical stamina. The symptoms of exhaustion are typically accompanied by other symptoms such as sleeping problems, cognitive difficulties, emotional lability, increased sensitivity to stimuli (sounds, light, etc.), and increased sensitivity to further stress [7].

A number of rating scales have been developed to conform to the diagnostic criteria of exhaustion disorder [8–10]. KEDS [11] is a brief, symptom-focused rating scale with excellent sensitivity and specificity when comparing patients with exhaustion disorder to healthy control individuals. However, little is known about the performance of KEDS in other patient groups. Because the symptoms addressed in KEDS cover several domains and may occur in many psychiatric and somatic disorders, it can be presumed that they may also be reported to some extent by patients with other diagnoses.

The purpose of the present study was to test a newly-translated Danish version of KEDS in patients from three DOMs. We aimed to answer four questions. First, how are symptoms of exhaustion distributed in different patient groups, including patients with different diagnoses, men and women, and young and old patients? Second, are the symptom levels for exhaustion different in patients diagnosed with stress-related disorders compared to patients with other mental disorders and patients diagnosed with somatic disorders? Third, is KEDS useful for distinguishing between patients diagnosed with stress-related disorders and patients with other disorders, and may it as such be used for diagnostic purposes? Finally, is KEDS free of item bias, i.e. does the response to individual questions in the rating scale differs systematically between different patient groups?

## Methods

### Patients

For a period of nine months (November 2015 to June 2016), patients who visited three DOMs (A, B, and C) in Odense, Copenhagen, and Herning in Denmark were consecutively invited to participate in the study. The inclusion criteria were patients who were Danish-speaking and visiting the clinic for the first time. Patients who did not speak Danish, and hence were unable to understand the questions in KEDS, were not included. Patients who were not visiting the clinic for the first time, such as those who were there for further counselling or treatment or who had already received a diagnosis, were also excluded. Seeking compensation for occupational injuries may influence the responses to questions in KEDS. Hence, we also excluded patients who were visiting the clinic for the purpose of obtaining a specialist statement, because these statements are used for assessing a worker’s right to compensation. Patients completed KEDS before the consultation and before they were informed about their diagnosis. At DOM A and B, patients completed KEDS upon entering the DOM, while at DOM C, the patients received the rating scale by mail and were asked to complete it before going to the DOM.

The symptoms of exhaustion were measured with KEDS [11]. KEDS was translated from Swedish to Danish and administered to 12 patients to test the comprehensibility (data from this test are not part of the main study). Based on the test, the text was revised, translated back to Swedish, and compared to the Swedish KEDS before being finally approved. The approved KEDS was used in the present study. The sum score (0–54) was used as a measure of exhaustion.

### Statistical methods

To compare the mean sum scores between patient groups, we used a mixed model that included group and gender as categorical variables, age as a continuous variable, and DOM as a repeated measure to account for the correlation between patients measured at the same DOM. Mixed models were estimated using the mixed procedure in the IBM SPSS Statistics software package. Pairwise differences in the KEDS sum scores of different patient groups were statistically tested using the estimated marginal means option. *P*-values were Bonferroni-corrected for multiple comparisons. The sensitivity and specificity of the KEDS sum score with regard to stress-related disorders were used to express the ability of KEDS to distinguish between patients diagnosed with stress-related disorders and patients diagnosed with somatic disorders. Sensitivity and specificity were calculated using the ROC procedure in the IBM SPSS Statistics package. IBM SPSS Statistics version 24 was used throughout these analyses. Two-tailed Bonferroni-adjusted *P* < 0.05 was used as the criterion of significance.

Differential item functioning (DIF) was used to assess item bias. In brief, an item shows DIF if different groups differ systematically in their response to the item after matching for the underlying characteristics that the item is intended to measure (in this study, exhaustion disorder). DIF is a necessary, but not a sufficient, condition for bias [12]. We evaluated DIF against patient groups (stress-related disorders versus somatic disorders), gender, and age (dichotomized at the median) using ordinal logistic regression [12]. The criteria for DIF were that the association between an item and a background variable such as gender was significant (*P* < 0.05, Bonferroni-adjusted) and of a sufficient magnitude. In line with a previous study [13], the criterion for sufficient magnitude was that the background variable explained at least an additional 2% of the item variance. SAS version 9.4 was used for the DIF analysis.

## Results

### Data collection

Fully or partly completed KEDS rating scales were received from 748 patients, from a total of 2500 eligible care-seeking patients at three occupational clinics (participation rate 30%). Fifty patients were subsequently excluded from the statistical analyses because of missing information (diagnosis, gender, age, or one or more missing responses in items of the KEDS scale). The remaining 698 patients comprised 461 women (66%) and 237 men (34%). The mean age was 46.2 years (range 19–82 years). Table 1 shows the distribution of diagnoses sorted according to the number of patients. A total of 89 different diagnostic categories (three-figure diagnostic codes) are represented, but most of them by fewer than five patients. The most prevalent diagnostic category was F43 (reaction to severe stress and adjustment disorders) with 217 patients (31%), followed by Z56 (problems related to employment and unemployment) with 99 patients (14%), and Z04 (examination and observation for other reasons) comprising 41 patients (5.9%; Table 1). It should be noted that diagnoses in the Z-block (Z[00–99]) are not codes for diseases, but are ‘provided for occasions when circumstances other than a disease, injury or external cause classifiable to categories A00-Y89 are recorded as “diagnoses” or “problems”’ [14].

**Table 1:** Diagnoses in 698 patients sorted according to frequency^a^

ICD-10 code	*N*	Pct.	Description	Diagnosis-based patient group		
				Stress-related disorders	Other psychiatric disorders than stress	Somatic disorders
F43	217	31.1%	Reaction to severe stress and adjustment disorders	x		
Hereof:						
F43.2	103	11.3%	Adjustment disorder	x		
F43.9	89	9.7%	Reaction to severe stress, unspecified	x		
F43.8	14	1.5%	Other reactions to severe stress	x		
F43.1	6	0.7%	Post-traumatic stress disorder	x		
F43.0	4	0.4%	Acute stress reaction	x		
F43	1	0.1%	Reaction to severe stress and adjustment disorders	x		
Z56	99	14.2%	Problems related to employment and unemployment		x	
Z04	41	5.9%	Examination and observation for other reasons			x
M75	34	4.9%	Shoulder lesions			x
M54	24	3.4%	Dorsalgia			x
F32	18	2.6%	Major depressive disorder, single episode		x	
F33	16	2.3%	Major depressive disorder, recurrent		x	
Z73	15	2.1%	Problems related to life-management difficulty			x
M77	14	2.0%	Other enthesopathies			x
M79	14	2.0%	Other soft tissue disorders, not classified elsewhere			x
Z10	11	1.6%	Routine general health check-up of defined subpopulation			x
J45	10	1.4%	Asthma			x
G56	9	1.3%	Upper-limb mononeuropathies			x
J44	9	1.3%	Other chronic obstructive pulmonary disease			x
M53	8	1.1%	Other dorsopathies			x
L24	7	1.0%	Irritant contact dermatitis			x
L30	6	0.9%	Other dermatitis			x
M23	6	0.9%	Internal derangement of knee			x
M51	6	0.9%	Other intervertebral disc disorders			x
M65	6	0.9%	Synovitis and tenosynovitis			x
F41	5	0.7%	Other anxiety disorders		x	
J31	5	0.7%	Chronic rhinitis, nasopharyngitis, and pharyngitis			x
M18	5	0.7%	Arthrosis of first carpometacarpal joint			x
M62	5	0.7%	Other muscle disorders			x
R05	5	0.7%	Cough			x
Total (including diagnoses not listed above)				217	143	338

^a^The total number of patients is 698. The sum of the column N is less than 698 because only diagnostic categories with five patients or more are listed

### KEDS sum scores in different patient groups

Three groups were formed to examine the distribution of the KEDS sum scores in different patient groups. The groups were ‘stress-related diagnoses’, ‘psychiatric diagnoses other than stress’, and ‘somatic diagnoses’. The ‘stress-related diagnoses’ group included patients with an F43 diagnosis. The ‘psychiatric diagnoses other than stress’ group included patients with F-diagnoses other than F43 (mainly depression diagnoses: F32 and F33) and patients with problems related to employment and unemployment (Z56). Patients with a Z56 diagnosis were included in this group because it is the standard practice at DOMs to use this diagnosis for patients with milder symptoms who do not meet the criteria for a mental disorder. The ‘somatic diagnoses’ group included patients with all diagnostic codes other than F-codes and Z56.

The mean KEDS sum scores and standard deviations for the three diagnosis-based patient groups, as well as for the selected subgroups, are presented in Table 2. The difference in the KEDS sum scores between patients with stress-related diagnoses, patients with psychiatric diagnoses other than stress, and patients with somatic diagnoses were all statistically significant. The largest mean KEDS sum score was observed in patients with stress-related diagnoses (29.3), while the mean KEDS sum score was intermediate in the group with psychiatric diagnoses other than stress (25.9) and was lowest in patients with somatic diagnoses (17.6; Table 2). An examination of the subgroups revealed that patients diagnosed with major depressive disorder had a high mean sum score (31.4) comparable to that of patients with stress-related diagnoses (Table 2). The mean KEDS sum score of patients with the Z56 diagnosis (23.5) was between that of patients with stress-related diagnoses and patients with somatic diagnoses (Table 2).

**Table 2:** Mean KEDS sum scores in patients with different diagnoses (main groups and subgroups)

Diagnosis-based patient group	ICD-10 codes	All		Men		Women	
		*N*	Mean (SD)	*N*	Mean (SD)	*N*	Mean (SD)
*Main group: stress-related diagnoses*	F43	217	29.3 (8.0)^e^	50	28.8 (8.7)	167	29.4 (7.8)
Subgroups:							
- *Adjustment disorder*	F43.2	103	29.9 (7.2)^f^	20	32.1 (7.7)	83	29.4 (7.0)
- *Reaction to severe stress, unspecified*	F43.9	89	27.9 (8.2)^f^	19	24.9 (7.7)^k^	70	28.6 (8.3)^k^
- *Other stress-related disorders*	Other F43.x^a^	25	31.8 (9.5)^f^	11	29.6 (10.0)	14	32.6 (8.9)
*Main group: psychiatric diagnoses other than stress*	F (except F43) + Z56	143	25.9 (9.5)^e^	34	24.3 (8.9)	109	26.3 (9.6)
Subgroups:							
- *Major depressive disorder*	F32 + F33	34	31.4 (8.1)^f^	10	28.4 (4.7)^l^	24	32.6 (8.9)^l^
- *Problems related to employment and unemployment*	Z56	99	23.5 (9.0)^g,i^	20	21.2 (10.0)	79	24.1 (8.7)
- *Other psychiatric disorders*	Other codes than those above^b^	10	30.1 (10.5)^h^	4	29.8 (4.9)	6	30.3 (13.6)
*Main group: somatic diagnoses*	All somatic codes^c^	338	17.6 (10.8)^e^	153	15.6 (9.7)^l^	185	19.2 (11.5)^l^
Subgroups:							
- *Examination and observations for other reasons*	Z04	41	18.7 (12.6)^g^	19	13.9 (8.4)^k^	22	22.9 (14.2)^k^
- *Shoulder lesions*	M75	34	17.5 (10.2)^g,j^	16	17.9 (11.9)	18	17.1 (8.7)
- *Dorsalgia*	M54	24	18.2 (9.4)^g^	12	18.2 (9.7)	12	18.2 (9.5)
- *Other somatic disorders*	Other codes than those above^d^	239	17.3 (10.7)^g,i,j^	106	15.3 (9.5)	133	18.9 (11.1)

Notes: ^a^F43 codes other than F43.2 and F43.9. ^b^F-codes other than F43, F32, and F33. ^c^Other codes than F-codes and Z56. ^d^All codes except F, Z56, Z04, M75, and M54. ^e-j^Statistically significant differences in mean KEDS sum score in pairwise comparison of patient groups in mixed models adjusted for sex and age as follows: ^e^The main groups of ‘stress-related diagnoses’, ‘psychiatric diagnoses other than stress’, and ‘somatic diagnoses’ differ from each other (all *P* < 0.001). ^f^Different from Z56, Z04, M75, M54, and ‘other somatic disorders’ (all *P* < 0.01). ^g^Different from F43.2, F43.9, ‘other stress-related disorder’, and F32 + F33 (*P* < 0.01). ^h^Different from M75 and ‘other somatic disorders’ (both *P* < 0.05). ^i^Significant difference between Z56 and ‘other somatic disorders’. ^j^Different from ‘other psychiatric diagnoses’. Bonferroni-correction was used in all post-hoc tests. ^k^*P* < 0.05 for the difference between men and women; ^l^*P* < 0.01 for the difference between men and women

In general, women scored higher than men, and the difference was statistically significant in the group of patients with somatic diagnoses (women 19.2 versus men 15.6, *P* < 0.01). A statistically significant difference between men and women was also observed for specific diagnoses, i.e. for F43.9, F32-F33, and Z04 (Table 2). The association between age and KEDS scores was not statistically significant.

To summarize the results: the mean KEDS sum score is higher in patients with psychiatric diagnoses (including diagnoses of stress-related disorders and major depression) than in patients with somatic diagnoses and is intermediate in patients assigned the Z56 diagnosis.

### Sensitivity and specificity with regard to stress-related diagnoses

**Fig. 1:**
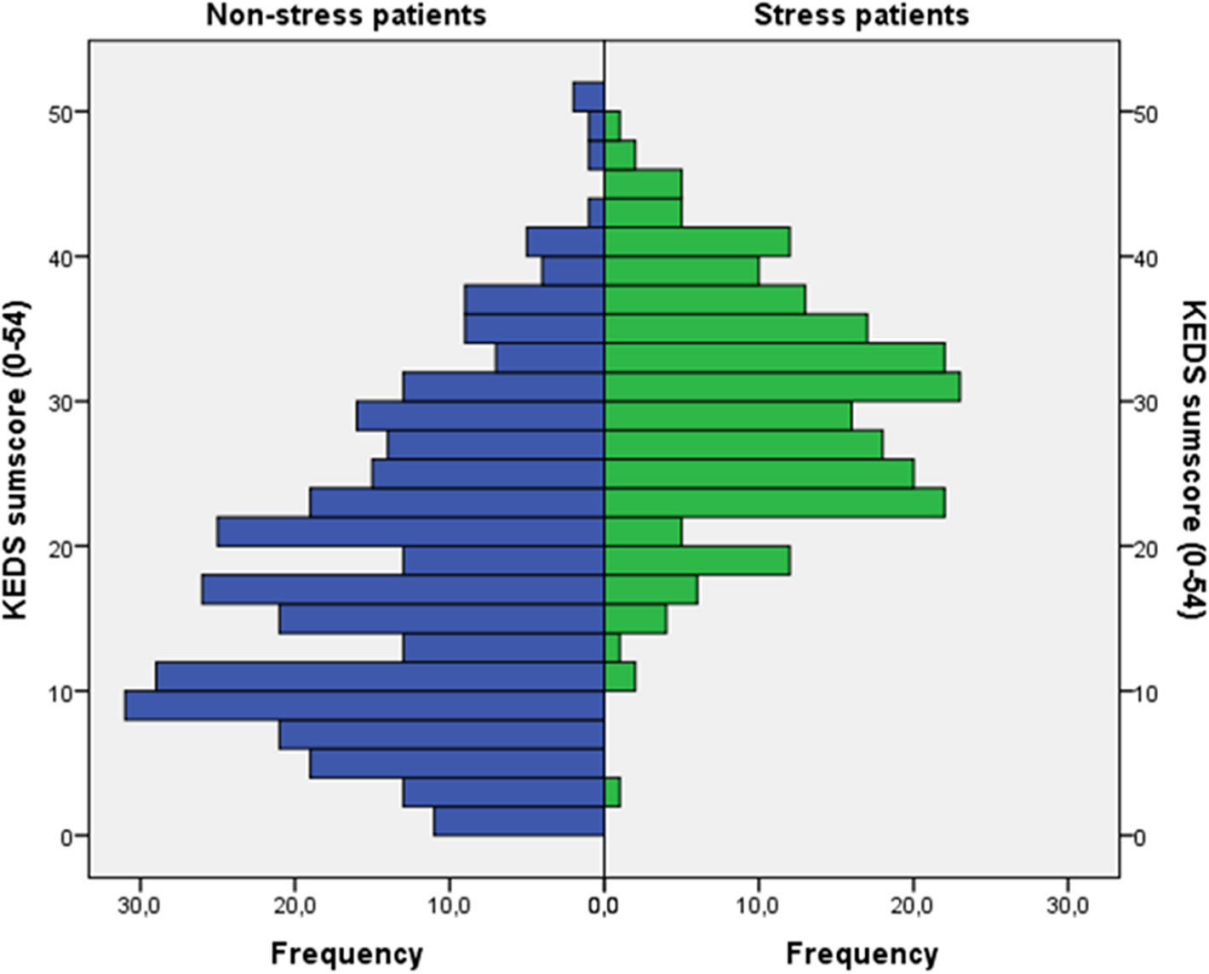
Distribution of KEDS scores (0–54) in 338 patients with somatic diagnoses (blue) and 217 patients with stress-related diagnoses (green)

Figure 1 presents the distribution of KEDS sum scores in patients with stress-related diagnoses and somatic diagnoses, respectively. The sensitivity and specificity of the KEDS sum score with regard to the identification of patients with stress-related diagnoses are shown in Table 3. The data indicate that when sensitivity is high, specificity is relatively low, and vice versa. As can be seen in Table 3, a cut-off score close to 19 [11] produces a reasonable sensitivity of around 90% (lower in men than in women), but the specificity is only 50–60%.

**Table 3:** Sensitivity and specificity of KEDS (217 patients with stress-related diagnoses versus 338 patients with somatic diagnoses)

KEDS sum score	All		Men		Women	
	Sensitivity	Specificity	Sensitivity	Specificity	Sensitivity	Specificity
16.5	0.95	0.51	0.90	0.59	0.96	0.44
18.5	0.89	0.57	0.82	0.65	0.92	0.50
21.5	0.86	0.66	0.80	0.75	0.87	0.58
25.5	0.66	0.76	0.66	0.85	0.67	0.68
30.5	0.46	0.87	0.48	0.93	0.45	0.82

### Differential item functioning

None of the KEDS items showed significant DIF when evaluated against patient group (stress-related diagnoses versus somatic diagnoses), gender, or age.

### Symptoms of exhaustion by diagnosis

**Fig. 2:**
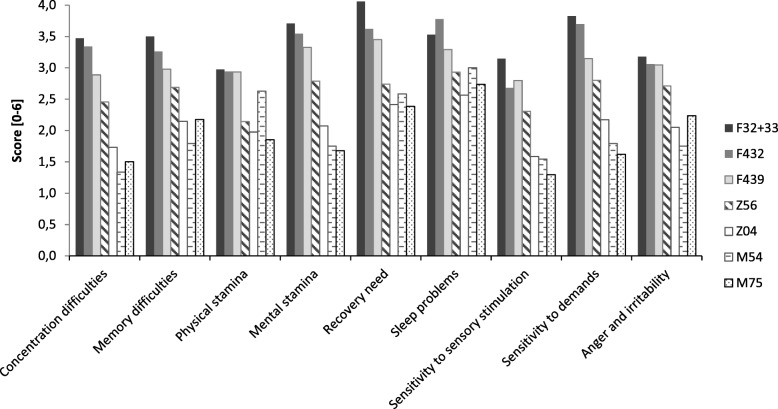
Mean KEDS item scores (0–6) for selected ICD-10 three-figure codes (see Table 1). Stress-related diagnoses: F43.2 (adjustment disorder), F43.9 (reaction to severe stress, unspecified). Psychiatric diagnoses other than stress: F32 + F33 (major depression), Z56 (problems related to employment and unemployment). Somatic diagnoses: Z04 (examination and observations for other reasons), M75 (shoulder lesions), M54 (dorsalgia)

The results suggest that symptoms of exhaustion are distributed unevenly in different patient groups. To further explore the distribution of symptoms of exhaustion in patient groups, the mean score for the nine symptoms of exhaustion in KEDS are presented in Fig. 2 for different diagnoses for which there are 20 patients or more. The results reveal that, in general, patients with psychiatric diagnoses (F43.2, F43.9, or F32-F33) score higher than patients with somatic diagnoses and patients with the Z56 diagnosis on all nine symptoms. The least contrast between groups is observed for sleep problems (see Fig. 2). Based on the sum scores, the data in Fig. 2 also confirm the above finding that the Z56 patient group is an ‘intermediate’ group with regard to symptoms of exhaustion. With a few exceptions (sleep problems, physical stamina difficulties, and recovery needs), the patients in this group score somewhere between patients with psychiatric diagnoses and patients with somatic diagnoses with regard to specific symptoms of exhaustion.

## Discussion

The Danish version of KEDS was tested in 698 patients from three Danish DOMs. The findings of this study are, firstly, that the level of the symptoms of exhaustion as assessed by KEDS is similar in patients with stress-related diagnoses and patients with major depressive disorder and secondly, that both of these groups report more symptoms of exhaustion than patients with somatic diagnoses and patients with ‘problems with employment and unemployment’ (diagnosis code Z56). A third finding is that the Z56 group also reports more symptoms of exhaustion than patients with somatic diagnoses. This finding corroborates the general experience that Z56 patients often display mild signs of a mental disorder but do not meet the criteria for the disorder. The examination of the distribution of the nine specific symptoms addressed by KEDS showed that patients with stress-related diagnoses and patients with major depressive disorder had higher levels of all symptoms than patients with somatic diagnoses. This result underscores that the symptoms of exhaustion addressed in KEDS are indeed characteristics of psychiatric disorders, although they are not very specific. The examination also revealed that the item addressing sleep problems was least able to differentiate between patients with psychiatric and somatic diagnoses. This is in accordance with the observation by Besèr et al. (2014), who found that sleep problems (together with anger and irritability) were associated with the weakest loading in the exploratory factor analysis when investigating the factor structure of the KEDS items.

With regard to the question of KEDS’ ability to differentiate between patients with stress-related diagnoses and patients with somatic diagnoses, the answer is negative, i.e. KEDS cannot be used for diagnostic purposes. Although the mean KEDS sum score of patients with stress-related diagnoses was significantly higher than that of patients with somatic diagnoses, the specificity was deemed to be too low at the cut-off score of 19 suggested by Besèr et al. [11] for the KEDS sum score to be useful for diagnostic purposes. Lastly, the statistical analysis established that none of the nine items on the KEDS scale showed DIF with regard to the patient group (stress-related disorder compared to somatic disorder), gender, or age. This means that the differences in the KEDS sum scores between these groups are not driven by single items to any significant extent, thus providing the answer to the fourth and final research question.

KEDS is a relatively new rating scale and there is only a limited amount of data available to which we can compare our results. In the validation study of KEDS, Besèr et al. [11] found a mean KEDS score of 29.9 (SD = 6.6) in 200 patients with exhaustion disorder and a mean KEDS score of 6.2 (SD = 5.3) in 117 healthy individuals. The mean sum scores of patients with stress-related diagnoses (29.3; SD = 8.0) and major depressive disorder (31.4, SD = 8.1) in this study are similar to the mean sum score of patients with exhaustion disorder in their study. However, it is noteworthy that the mean KEDS sum score of patients with somatic diagnoses was relatively high compared to that of the healthy individuals in the study by Besèr et al. [11]. This is not surprising, considering that it is well-known that various non-psychiatric diseases are associated with increased levels of fatigue [15–21]. Moreover, it has been shown that symptoms of fatigue are prevalent in primary care patients [22, 23] and that somatic disease is associated with increased levels of mental fatigue among the general population [24]. Nevertheless, the results in this study suggest that pronounced exhaustion is a characteristic of patients with psychiatric disorders, including stress-related disorders.

The earlier phases of exhaustion disorder are amenable to preventive measures [25]. The high level of symptoms of exhaustion in the Z56 patients observed in this study suggests that KEDS might be used to identify an early prodromal phase of the exhaustion disorder. A high level of symptoms of exhaustion could serve as a warning sign and may lead the doctor to inquire about possible underlying causes. This may lead to a treatment that could help the patient and alleviate the symptoms.

Moreover, symptoms of exhaustion also persist for a long time in patients with stress-related disorders [26]. Since mental and physical exhaustion will affect the patient’s daily functioning, it is plausible that the manifestation of symptoms of exhaustion may interfere with the patient’s compliance with treatment or may complicate the return to work from a sickness-related absence [27]. Hence, KEDS could also be useful in assessing symptoms of exhaustion in secondary prevention as well as in determining when patients are ready to return to work.

This study has strengths as well as limitations. A strength is that it investigates symptoms of exhaustion in a clinical population [28]. Furthermore, it is also a strength that the patients were recruited from three different DOMs, which increases the generalizability of the findings to patients in other DOMs. However, the low overall response rate must be mentioned as one of the limitations of the study. This might have influenced the mean score of the different patient subgroups to some extent, because highly exhausted patients are more likely to refuse to participate in the study. The mean KEDS sum score of patients with stress was, however, almost identical to the mean score of patients with exhaustion disorder in the study by Besèr et al. [11], which we interpret to indicate that this effect was not very strong. Another limitation is that information about the patients who chose not to participate was not collected in the study. Thus, a drop-out analysis could not be made. It is also a limitation that the classification of patients was based solely on the diagnosis assigned at the DOM. Hence, patients diagnosed with a somatic disorder may also have a stress-related disorder, depression, or another psychiatric disorder. However, double diagnoses were recorded and constituted only a negligible fraction. Therefore, we believe that the possible contribution from undetected severe psychiatric disorders does not explain the level of symptoms of exhaustion in patients with somatic diagnoses. Finally, it is a limitation that we did not include a non-patient comparison group in the current study.

## Conclusions

Patients with stress-related diagnoses (ICD-10 diagnostic codes F43) and patients diagnosed with major depression (F32 + F33) reported stronger symptoms of exhaustion as assessed by KEDS than a diverse group of patients with various somatic diagnoses. However, the specificity of the KEDS sum scores was too low for KEDS to be useful for differential diagnostics. On average, and regarding both the total KEDS score and the scores on individual KEDS items, women tended to select the more severe response alternatives (i.e., score higher). Patients without a disorder diagnosis but with problems due to employment or unemployment (Z56) reported a level of symptoms between that of patients with psychiatric diagnoses and patients with somatic diagnoses. This might indicate a mild exhaustion state that warrants attention from the doctor. The ability of KEDS to detect mild states of exhaustion, which are possible pre-stages to severe stress-related disorders, needs to be further investigated.

## Abbreviations

DIF: differential item functioning
DOM: Department of occupational medicine
ICD: International Classification of Diseases
KEDS: Karolinska Exhaustion Disorder Scale

## References

[1] Carder M, McNamee R, Turner S, Hodgson JT, Holland F, Agius RM (2013). Time trends in the incidence of work-related mental ill-health and musculoskeletal disorders in the UK. Occup Environ Med.

[2] Åsberg M, Grape T, Krakau I, Nygren Å, Rodhe M, Wahlberg A, Währberg P (2010). Stress som orsak til psykisk ohälsa. Läkartidningen.

[3] Gradus JL, Bozi I, Antonsen S, Svensson E, Lash TL, Resick PA, Hansen JG (2014). Severe stress and adjustment disorder diagnoses in the popuation of Denmark. J Traumatic Stress.

[4] Arbejdstilsynet [The Danish Working Environment Authority]: Årsopgørelse - Anmeldte erhvervssygdomme 2014 [Annual statement - notified occupational diseases 2014]. 2015:1–30.

[5] Dansk Selskab for Arbejds- og miljømedicin [Danish society for occupational and environmental medicine]: Medicinsk Teknologi Vurdering af Behandling af arbejdsrelateret Stress. 2012: 1–51.

[6] Pedersen CB, Mors O, Bertelsen A, Waltoft BL, Agerbo E, McGrath JJ, Mortensen PB, Eaton WW (2014). A comprehensive nationwide study of the incidence rate and lifetime risk for treated mental disorders. JAMA Psychiatry.

[7] Socialstyrelsen [Swedish National Board of Health and Welfare].: Utmattningssyndrom - stressrelaterad psykisk ohälsa [Exhaustion disorder - stress-related mental ill-health]. Stockholm; 2003: 1–88.

[8] Glise K, Hadzibajramovic E, Jonsdottir IH, Ahlborg G (2010). Self-reported exhaustion: a possible indicator of reduced work ability and increased risk of sickness absence among human service workers. Int Arch Occup Environ Health.

[9] Saboonchi F, Perski A, Grossi G (2013). Validation of Karolinska exhaustion scale: psychometric properties of a measure of exhaustion syndrome. Scand J Caring Sci.

[10] Persson R, Österberg K, Viborg N, Jönsson P, Tenenbaum A (2016). The Lund University checklist for incipient exhaustion–a cross–sectional comparison of a new instrument with similar contemporary tools. BMC Public Health.

[11] Beser A, Sorjonen K, Wahlberg K, Peterson U, Nygren Å, Åsberg M (2014). Construction and evaluation of a self rating scale for stress-induced exhaustion disorder, the Karolinska exhaustion disorder scale. Scand J Psychol.

[12] Zumbo BD: A handbook on the theory and methods of differential item functioning (DIF): logistic regression modeling as a unitary framework for binary and Likert-type (ordinal) item scores. In*.* Ottawa, ON; 1999: 1–57.

[13] Bjorner JB, Pejtersen JH (2010). Evaluating construct validity of the second version of the Copenhagen psychosocial questionnaire through analysis of differential item functioning and differential item effect. Scand J Public Health.

[14] World Health Organization: ICD-10 Version:2010 (https://icd.who.int/browse10/2016/en). Accessed 28 Feb 2019.

[15] Langballe EM, Innstrand ST, Hagtvet KA, Falkum E, Aasland OG (2009). The relationship between burnout and musculoskeletal pain in seven Norwegian occupational groups. Work.

[16] Hagen EM, Svensen E, Eriksen HR, Ihlebæk CM, Ursin H (2006). Comorbid subjective health complaints in low back pain. Spine.

[17] Power DJ, Badley EM, French MR, Wall AJ, Hawker GA (2008). Fatigue in osteoarthritis: a qualitative study. BMC Musculoskelet Disord.

[18] Breslin E, van der Schans C, Breukink S, Meek P, Mercer K, Volz W, Louie S (1998). Perception of fatigue and quality of life in patients with COPD. Chest.

[19] Small S, Lamb M (1999). Fatigue in chronic illness: the experience of individuals with chronic obstructive pulmonary disease and with asthma. J Adv Nurs.

[20] Theander K, Unosson M (2004). Fatigue in patients with chronic obstructive pulmonary disease. J Adv Nurs.

[21] Janson C, de Backer W, Gislason T, Plaschke P, Björnsson E, Hetta J, Kristbjarnarson H, Vermeire P, Boman G (1996). Increased prevalence of sleep disturbances and daytime sleepiness in subjects with bronchial asthma: a population study of young adults in three European countries. Eur Respir J.

[22] Lewis G, Wessely S (1992). The epidemiology of fatigue: more questions than answers. J Epidemiol Community Health.

[23] Kroenke K, Spitzer RL, Williams JBW, Linzer M, Hahn SR, deGruy IIIFV, Brody D (1994). Physical symptoms in primary care. Arch Fam Med.

[24] Watt T, Groenvold M, Bjorner JB, Noerholm V, Rasmussen NA, Bech P (2000). Fatigue in the Danish general population. Influence of sociodemographic factors and disease. J Epidemiol Community Health.

[25] Peterson U, Bergström G, Samuelsson M, Åsberg M, Nygren Å (2008). Reflecting peer-support groups in the prevention of stress and burnout: randomized controlled trial. J Adv Nurs.

[26] Glise K, Ahlborg G, Jonsdottir IH (2012). Course of mental symptoms in patients with stress-related exhaustion: does sex or age make a difference?. BMC Psychiatry.

[27] Spelten ER, Verbeek JHAM, Uitterhoeve ALJ, Ansink AC, van der Lelie J, de Reijke TM, Kammeijer M, de Haes JCJM, Sprangers MAG (2003). Cancer, fatigue and the return of patients to work—a prospective cohort study. Eur J Cancer.

[28] Grossi G, Perski A, Osika W, Savic I (2015). Stress-related exhaustion disorder – clinical manifestation of burnout? A review of assessment methods, sleep impairments, cognitive disturbances, and neuro-biological and physiological changes in clinical burnout. Scand J Psychol.

[29] Undervisnings- og forskningsministeriet [Ministry of Higher Education and Science]. Lov om videnskabsetisk behandling af sundhedsvidenskabelige forskningsprojekter [Act on Research Ethics Review on Health Research Projects], 2011.

[30] ISO/IEC: Information technology -- Security techniques -- Information security management systems -- Requirements. In*.* Geneva, Switzerland: The International Organization for Standardization (ISO), the International Electrotechnical Commission (IEC); 2013.

[31] Justitsministeriet [Ministry of Justice]: Bekendtgørelse om sikkerhedsforanstaltninger til beskyttelse af personoplysninger, som behandles for den offentlige forvaltning (BEK 528 af 14. juni 2000) [Executive order on security measures to protect personal data that are processed by the public administration]. Ministry of Justice; 2000.

